# The COVID-19 Pandemic and the Rental Market: Evidence From Craigslist

**DOI:** 10.1177/00027642211003149

**Published:** 2021-11

**Authors:** John Kuk, Ariela Schachter, Jacob William Faber, Max Besbris

**Affiliations:** 1 University of Oklahoma, Norman, OK, USA; 2 Washington University in St. Louis, St Louis, MO, USA; 3 New York University, NY, USA; 4 University of Wisconsin–Madison, Madison, WI, USA

**Keywords:** rental housing prices, Craigslist, neighborhoods, racial inequality, COVID-19 pandemic

## Abstract

Past research has demonstrated the racially and spatially uneven impacts of economic shocks and environmental disasters on various markets. In this article, we examine if and how the first few months of the COVID-19 pandemic affected the market for rental housing in the 49 largest metropolitan areas in the United States. Using a unique data set of new rental listings gathered from Craigslist and localized measures of the pandemic’s severity we find that, from mid-March to early June, local spread of COVID-19 is followed by reduced median and mean rent. However, this trend is driven by dropping rents for listings in Black, Latino, and diverse neighborhoods. Listings in majority White neighborhoods experience rent increases during this time. Our analyses make multiple contributions. First, we add to the burgeoning literature examining the rental market as a key site of perpetuating sociospatial inequality. Second, we demonstrate the utility of data gathered online for analyzing housing. And third, by reflecting on research that shows how past crises have increased sociospatial inequality and up-to-date work showing the racially and spatially unequal effects of the COVID-19 pandemic, we discuss some possible mechanisms by which the pandemic may be affecting the market for rental housing as well as implications for long-term trends.

The COVID-19 pandemic has affected almost every aspect of social life. In addition to the horrific loss of life, the economic effects of the crisis were precipitous and will undoubtedly be long-lasting. Shelter in place orders and fear of transmission reduced mobility in the United States substantially and staggering job loss suggests that there will be subsequent effects on both supply and demand in various markets. Fine-grained analyses of consumption and employment reveal that as local rates of infection rose, places experienced steep declines in spending (Chetty et al., 2020).

Within these trends, there is important heterogeneity. On the one hand, certain economic sectors, including suburban home ownership markets, have experienced rapid growth as wealthier, mostly White households attempted to flee pandemic hotspots (Quealy, 2020). In contrast, neighborhoods with higher levels of within-household crowding—where more residents are Black and/or Latino—experienced higher infection rates (Chang et al., 2021; Friedman & Rosenbaum, 2004; The Stoop, 2020). In addition to highlighting the disproportionate impact of COVID-19 across ethnoracial groups, both in terms of health (Bambino et al., 2020; Garcia et al., 2020; Kullar et al., 2020; Oppel et al., 2020; van Dorn et al., 2020) and economic effects (Brown, 2020; Smialek & Tankersley, 2020), these contrasting trends highlight the importance of studying various aspects of the housing market in order to understand the uneven economic impacts of COVID-19. More broadly, prior research on disasters suggests that the effects of COVID-19 on the housing market in general and rental market in particular are likely to be severe and deeply unequal; neighborhoods with more Black and Latino residents may suffer disproportionately (Faber, 2018b; Ortega & Taspinar, 2018).

In this article, we explore the effects of the first few months of the COVID-19 crisis in the United States on the market for metropolitan rental housing. We examine overall trends across metropolitan areas as well as test for distinct effects across neighborhoods differentiated by ethnoracial characteristics. Although our analysis focuses on the period between March and June 2020, we are motivated by the potential long-term consequences of changing rental market conditions. For example, lack of demand and declining economic activity in general may reduce the supply of affordable housing in Black and Latino neighborhoods in the long-term (Immergluck et al., 2016). In the aftermath of the Great Recession, a great deal of rental housing transitioned from being owned by individual, “mom and pop” landlords to large corporate landlords (Aalbers, 2019; Immergluck & Law, 2014; Wijberg et al., 2018)—especially in Black neighborhoods (Hwang, 2019). While the effects of this transition remain a subject of research, early work indicates that the financialization of the rental market and more corporate ownership of rental housing stock increases eviction rates (Raymond et al., 2016) and negatively impacts unit quality (Hwang, 2019). Indeed, if individual landlords lose their homes to foreclosure—a reasonable prediction given the trends in the economy precipitated by the COVID-19 crisis—more corporate control over the rental market is likely (Herbert et al., 2013). More generally, if past crises are predictive, turmoil in the housing markets could lead to more residential segregation (Hall et al., 2015) and lingering impacts on racial inequality in housing opportunity (Faber, 2018b, 2019a).

To understand the pandemic’s impact on the rental market, we assembled a data set on rental housing in the 49 largest metropolitan statistical areas (MSAs) gathered continuously throughout the crisis from Craigslist, the largest website for rental housing advertisements in the United States. We combine data on millions of geocoded rental listings with measures of each metro’s daily positive COVID-19 cases. We leverage descriptive analyses and two-way fixed effects regression models to estimate the relationship between rising COVID-19 cases and the volume of posts (i.e., the number of available rental units advertised) and the advertised rent of available units.

Overall, we find that, from mid-March to early June—a time period capturing the initial surge and first national peak of infections and deaths—an increase in the number of COVID-19 cases within an MSA is associated with reduced MSA median and mean rental prices. However, this overall relationship masks substantial racial heterogeneity. Specifically, we find that the negative correlation between price and new cases is driven by falling rental prices across listings in predominantly Black, Latino, and diverse neighborhoods. Conversely, for listings in predominantly White neighborhoods, newly reported cases across the metro are *positively* correlated with price. We find no significant effect of COVID-19 infection rates on listing volume. While we are limited in our ability to uncover the mechanisms by which rising COVID-19 cases might affect the rental market, our findings are consistent with other analyses indicating the economically, racially, and spatially disparate impact of COVID-19 (Borjas & Cassidy, 2020; Chetty et al., 2020; Gaynor & Wilson, 2020).

Although the effects of the COVID-19 pandemic will be long-lasting, these initial analyses carry important implications for understanding racial inequality in a rapidly changing and unprecedented social and economic catastrophe. Our contributions are therefore multiple. First, we add to a bourgeoning literature across the social sciences that examines the market for rental housing. Since the Great Recession, the share of Americans in the rental market has increased, particularly in urban areas. There is growing recognition that more theoretical, empirical, and policy-related research on the rental market is necessary. Second, we demonstrate the utility on data gathered online for analyzing housing. The housing market in general—and the rental market in particular—increasingly operate online and various tools at social scientists’ disposal can be used to create more fine-grained and up-to-date data sets (McLaughlin & Young, 2018). Third, we analyze the indirect effects of a particular kind of crisis. Research in economics and sociology has begun to assess the various, if not always obvious, costs of both immediate shocks, like floods, and more long-term transformations to the physical and social environment, like climate change, on the housing market (Desmet & Ross-Hansberg, 2015; Rhodes & Besbris, in press). Following this work, we examine how a global pandemic may be altering trends in the rental market. Furthermore, our analyses lead to other research questions about the sustained and racially unequal impact of COVID-19 on the housing market—key questions given the duration of the pandemic and the concomitant economic fallout.

## The Rental Market

Almost half of central city residents in the United States are renters, as are the majority of low-income households, non-White households, and immigrant households (Ellen & Karfunkel, 2016; JCHS, 2019). Renters are far more mobile than homeowners, have different rates of racial/ethnic segregation, and tend to be far more cost-burdened by their housing expenses (Desmond, 2018; Friedman et al., 2013; Immergluck et al., 2017; Myers & Park, 2019). The rental market also has different supply, demand, and costs than the market for home purchasing and renters—unlike buyers who are connected to sellers through a host of intermediaries (real estate agents, mortgage brokers, and appraisers) that provide information about neighborhoods and housing units (Besbris, 2016, 2020; Besbris & Faber, 2017; Korver-Glenn, 2018)—face a unique set of risks and choice constraints (DeLuca et al., 2013; Desmond & Perkins, 2016; Wegmann et al., 2017). Yet there remains little research on the rental market’s role in shaping metropolitan processes like neighborhood demographic change, residential and income segregation, or geographic variations in household finances (Schachter & Besbris, 2017). This gap is, in large part, due to the lack of up-to-date and accurate data on the market for rental housing. Indeed, administrative rental market data sets tend to update slowly and are often aggregated, meaning they summarize measures like rent across a large set of housing units—obtaining disaggregated data from sources like the American Community Survey and the American Housing Survey tends to be onerous and the data are still only collected annual or every other year.

Recent work has recognized the utility of internet platform-based proprietary data sets on the rental market. These data are “tantalizingly rich, detailed, and rare in their ability to describe the spot market in disaggregate form” (Boeing et al., 2020, p. 6). For example, advertisements for rental housing on Craigslist must contain advertised rent and a textual description of the rental unit (Besbris et al., in press; Kennedy et al., 2020). The vast majority also contain an exact address or easily geocoded location on a map embedded in the ad, pictures of the unit, and specific information about the pet policy, access to laundry services, the square footage, and the number of bedrooms and bathrooms (Boeing et al., 2021). Despite their advantages, propriety data sets are not perfect representations of the rental market writ large (Boeing, 2020). They should therefore be used in concert with existing sources of data to examine rental market trends.

## External Shocks to the Housing Market

Past work has predicted that risks of various sorts will reduce rents (see Frame, 1998) and while there remains a dearth of research on how the rental market is affected by events like hurricanes, floods, and climate change more generally, there is a core set of findings from work on sales markets that guide our analyses below. Hurricanes, for example, displace both renters and homeowners but renters, particularly ones utilizing housing vouchers and government subsidies, return to affected neighborhoods more slowly (Fussell & Harris, 2014). In terms of supply, properties for sale recover far more quickly than rental properties after Hurricanes. Environmental disasters also precipitate housing market volatility, particularly in poor, non-White neighborhoods—potentially requiring moratoria on evictions and foreclosures to prevent further drops in demand as well as rising homelessness and household financial strain (Y. Zhang & Peacock, 2009).

A robust finding across multiple postdisaster analyses of the housing market is that, if properties are damaged, prices decline in the immediate aftermath and likely in the longer term as well. Hurricane Sandy, for example, caused large declines in home values for flooded properties (Ortega & Taspinar, 2018). Properties that were not damaged but are in flood zones also experienced a sustained price penalty—demonstrating long-term effects of the disaster on real estate prices. Similarly, after Hurricane Andrew, properties in Florida that were not damaged themselves but proximate to damaged ones experienced sustained price penalties (Hallstrom & Smith, 2005). However, the effect of risk is not evenly felt across the market—the negative impact of climate-related disasters is stronger on low-priced homes (L. Zhang, 2016).

The COVID-19 pandemic is distinct from disasters in which property is damaged. However, there are clear mechanisms by which the market for both rental and purchased residential property could be affected. Shelter-in-place orders, for example, reduce the ability to search for and view available properties while rising infection rates may reduce homeseekers’ desire to search. Depressed demand could result in both falling prices as well as reduced inventory if landlords choose to withhold listings. A recent industry report on Manhattan’s property market found that the number of completed sales transactions was down over 50%, the median price dropped nearly 18%, and the number of signed contracts for purchased properties dropped 76% in the second quarter of 2020 compared with the same period in 2019 (Chen & Franklin, 2020). Overall, the number of properties listed in the first half of 2020 was 26% lower in Manhattan than in the first half of 2019. We might similarly expect reduced volume of listings in rental markets affected by COVID-19 as landlords cannot show their properties to prospective tenants or do not list their available units believing that demand will increase at a later point in time. Indeed, in both Manhattan and Brooklyn, new lease signings continued to fall through June 2020 and the vacancy rate of rental units climbed to levels not seen in over a decade (Haag, 2020). However, past work has shown that rental market indicators like listing volume, vacancy rates, and rental price remain relatively sticky during economic downturns and do not necessarily covary (Genesove, 2003; Lens, 2018; also see Gabriel & Nothaft, 2001).

Any effects of the pandemic are likely to be socio spatially unequal. U.S. metropolitan areas are defined by ethnoracial and socioeconomic segregation within and between neighborhoods (Krysan & Crowder, 2017; Massey & Denton, 1993; Quillian & Legrange, 2016; Reardon & Bischoff, 2011). As a result, neighborhoods with higher shares of poor and non-White households tend to be deprived of economic and institutional resources (Quillian, 2012; Sampson, 2012; Small & McDermott, 2006; Wilson, 1987) and their residents face various forms of exploitation, discrimination, and disadvantage (Besbris et al., 2015; Besbris et al., 2019; Desmond & Wilmers, 2019; Faber, 2019b; Quillian, 2014; Sharkey, 2013; Sharkey & Faber, 2014). Before, during, and after the Great Recession, more segregated neighborhoods experienced more subprime lending, more foreclosures, and an increase in the establishment of fringe financial institutions (Faber, 2018a, 2018b; Wyly et al., 2009). Additionally, poor and non-White neighborhoods tend to be far more vulnerable to environmental disasters (Faber, 2015; Fothergill & Peek, 2004; Freudenberg et al., 2009; Tierney, 2007) and recover more slowly relative to more advantaged places (Curtis et al., 2015; Elliott, 2015; Fussell, 2015; Pais & Elliott, 2008). Disasters also increase inequality across households within affected places (Howell & Elliott, 2019). This past work motivates us to examine if and how the COVID-19 pandemic has altered rental market dynamics in disparate ways across neighborhoods that vary by racial demographics.

## Data

Our rental market data come from Craigslist. Social scientists are increasingly recognizing the value of online data (Lazer & Radford, 2017; Salganik, 2017); because Craigslist is widely recognized as the dominant platform for today’s metropolitan rental housing market and the primary source of information for the vast majority of rental housing searches (Boeing & Waddell, 2017), we view it as a promising data source. While Craigslist cannot provide a census of all rental housing in the United States, it is more comprehensive and timelier than any other existing sources including the American Community Survey and the American Housing Survey (Boeing et al., 2020; Boeing & Waddell, 2017).

We designed a set of Python scripts to crawl the web and collect information from rental ads, including listing date, rent (price), and geo-location. We include all Craigslist sites that correspond to the 49 largest MSAs in the United States.^1^ In all MSAs, posters creating ads for rental housing are asked to supply a geographic location on a Google maps image from which we extract the listings’ geocode. Across our metro areas, 0.8% of all listings are missing a geocode and are thus excluded from all analyses presented here. We use the geocodes to assign each advertisement to a MSA and a Census tract within it using the sp package in R for spatial overlay.

The Python scripts, which have been running continuously since late May, 2017, capture each unique listing posted in each MSA. The scripts revisit each MSA Craigslist site twice per week, and check to see whether each currently posted listing is new, in which case all information will be scraped, or if the listing is a repeat from the previous week, which is also noted in the database. We are therefore able to track how long listings remain on the market, as well as collect data on new listings each week. By visiting each site twice a week, we miss listings that are posted and removed within 3 to 4 days; however, since landlords are free to post listings at any time, this missing data should not bias any of our analyses. From March 1 of 2020 through June 1, 2020, we collected 2,449,753 listings across all 49 MSAs. We eliminate listings missing geocodes for a final data set of 2,418,162 geocoded listings.^2^ We then merge our data with 2018 ACS 5-year pooled data on neighborhood (e.g., tract) racial/ethnic composition, several indicators of socioeconomic status, and other neighborhood characteristics relevant to rental market dynamics. We capture COVID-19 positive cases over time at the metro level using data from Social Explorer.^3^ Our analyses are constrained by the lack of systematic data on COVID-19 cases at the sub-MSA level (i.e., zip code or tract).

## Method

We begin by examining descriptive changes in rents over time, starting March 1 (i.e., just before a national pandemic was declared on March 13, 2020) and through June 1st. We examine trends by MSA as well as in the aggregate given the differential impact of COVID-19 across MSAs and over time.

Next, we leverage variation over time within MSAs to estimate the relationship between new positive COVID-19 cases on listed rental unit volume and prices in the same MSA. Specifically, we estimate two-way fixed effects models that control for both time and time-invariant characteristics of MSAs that may drive rent prices and/or listing volume. While our goal is to isolate the relationship between COVID-19 cases and our market outcome measures, we do not claim to be identifying causal effects in this preliminary analysis; rather, we limit our interpretation to identifying potential relationships/associations which should motivate further inquiry. We use logged COVID-19 cases given the exponential nature of the pandemic.^4^ The unit of analysis is MSA-week. Our sample is a fully balanced panel of 49 MSAs measured each week for 12 weeks (i.e., 588 metro-weeks).^5^ We aggregate our outcome variables and covariates at the MSA level for each week.

**Figure A1.**
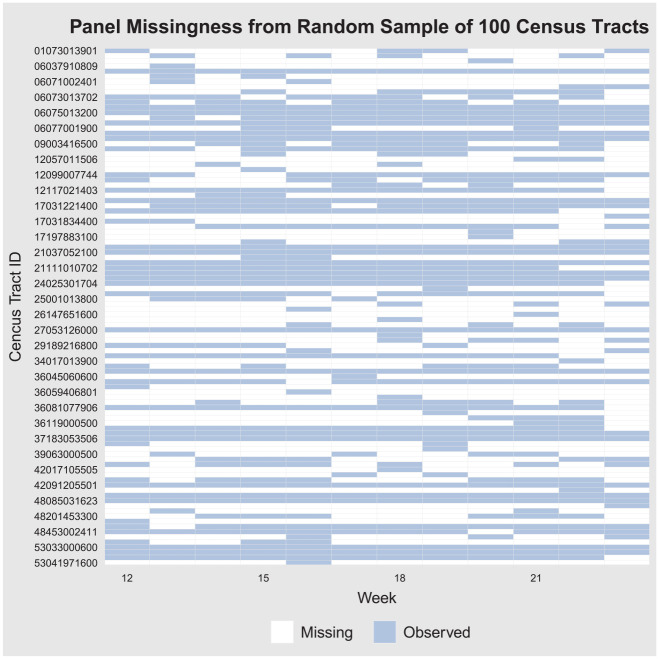
Panel missingness in Craigslist data from random sample of 100 census tracts.

Next, we test for heterogeneity in the effects of COVID within MSAs based on neighborhood race/ethnicity. To do so, within each metro we calculate average rental prices among listings located in four types of neighborhoods (White, Black, Latino, and diverse) in each MSA by week. We define the neighborhood type by the majority enthnoracial group in the Census tract. We classify neighborhoods that do not have a group population higher than 50% of the residents as diverse neighborhoods.^6^ We use this aggregation method rather than switching our unit of analysis to Census tracts because many tracts do not experience a high enough listing volume to calculate weekly price changes. See Figure A1, Appendix A, for details.^7^

The model to estimate the effect of COVID-19 cases is the following:

$$Y_{mt}=\;\beta \cdot \;\;logCOVID19cases_{mt}+\alpha_{m}+\;\delta_{t}+\epsilon_{it}$$

*Y_it_* is the outcome variable at MSA *m* and week *t. logCOVID19cases_mt_* indicates the natural logarithm of new COVID19 cases in MSA *m* and week *t*. The model includes MSA fixed effects ($\alpha_{m}$) and week fixed effects ($\delta_{t})$. For models estimating the differential effect by neighborhood types, we add the neighborhood type indicator and an interaction term between logged COVI19 cases and the neighborhood type variable. Standard errors are clustered at the MSA level.

## Results

**Figure 1.**
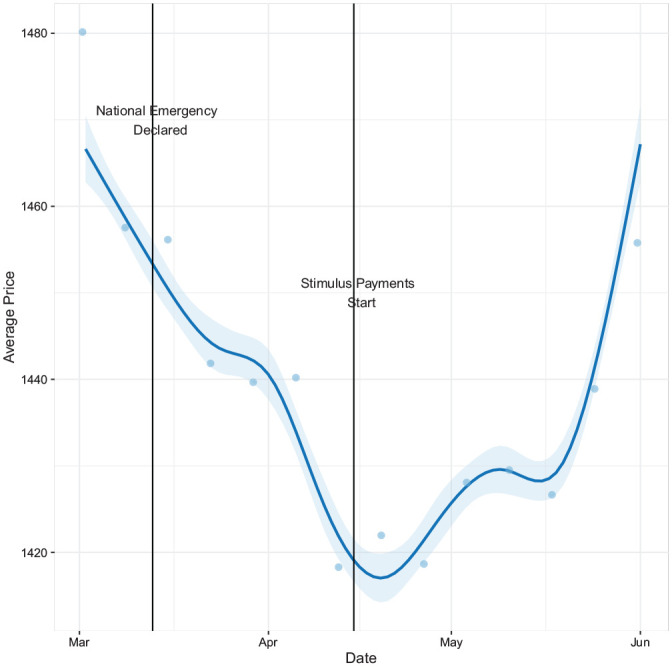
Trend of average rental price. *Note*. The figure displays the trend of average rental price of our whole Craigslist sample between March 1, 2020, and June 1, 2020. The dots indicate binned average price. The smooth line and confidence intervals are derived from using generalized additive models (GAM).

We begin by examining changes over time in rental price by averaging across all listings in all MSAs. Figure 1 shows a clear dip in asking rental prices from March through mid-April when the federal stimulus payments began on April 15 and COVID-19 cases had their first peak in many parts of the United States. Average rental prices then began to rebound, and by mid-June had almost fully recovered. These trends are similar to other economic data documenting sharp initial dips (Leatherby & Gelles, 2020), and partial recoveries starting in mid-April (Chetty et al., 2020).

**Figure 2.**
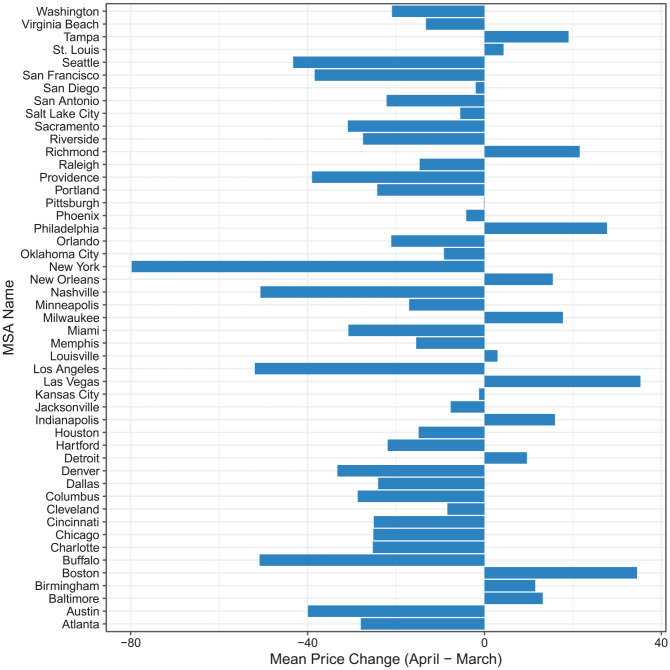
Change in average rental price between March and April by MSA. *Note*. The bars represent the change in average rental price for each MSA between March and April. The values are calculated by subtracting the average rental price of April from the average rental price of March. Negative values indicate there is an average price drop between March and April. MSA = metropolitan statistical area.

When we disaggregate changes in rental prices by MSA between March and April, we see distinct trajectories. As shown in Figure 2, while most MSAs experienced a decrease in average rental price, about one quarter experienced an increase in average price. While we do not have enough data to test potential explanations for this variation, some of these MSAs were experiencing the continuation of secular positive trends in rents before local outbreaks. It is also possible that these MSAs had characteristics that were particularly desirable among more mobile households during the first wave of the pandemic in the United States—such as lower density—or residents of these MSAs may have simply presumed that the local effects of COVID-19 would not be as severe relative to early epicenters like Seattle and New York. In other words, MSA-specific mobility patterns and other local contextual factors may contribute to variation in rental market trends during the pandemic. We also see variation among MSAs with net decreases in price; some, such as New York, which was the epicenter of the U.S. outbreak during this time, show a much larger drop in price relative to other areas. While more time and data are needed to understand the causes of this heterogeneity, Figure 2 highlights the differential impact of COVID-19 across geographies.

**Figure 3.**
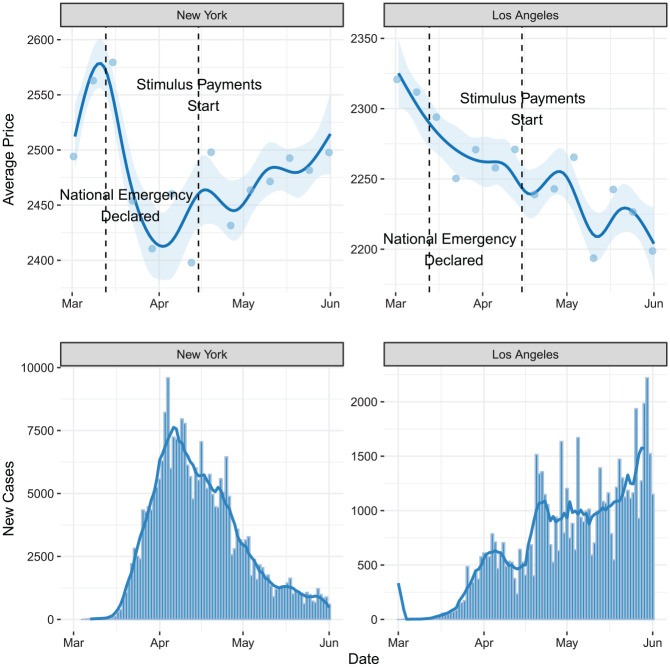
Trends of average rental price (above) and new COVID-19 cases (below) in New York and Los Angeles. *Note*. The figure above presents the average price trend of listings in New York and Los Angeles metropolitan statistical area. The bar plot below displays the number of new COVID-19 cases for each day. The blue trend line represents the 7-day average trend of new cases.

Next, to better understand whether MSA-level heterogeneity in rental price trends is tied to the differential effects of COVID-19, we examine two of the largest MSAs in our sample, New York City and Los Angeles. Indeed, as shown in Figure 3, we observe different price trends over time, which correspond with positive COVID-19 case numbers. In Los Angeles, where cases continued to rise throughout our period of analysis, we see a corresponding decrease in average listing price over time. In contrast, the plots for the New York more closely correspond with the overall averages presented in Figure 1, namely, a sharp decline in prices followed by a steady though partial recovery. Rental prices in New York reached their lowest levels at around the same time as the MSA’s new COVID-19 case count reached its peak. While not all MSAs look like either New York City or Los Angeles, they both illustrate how heterogeneity in COVID-19 case rates helps explain differential rental market trends.

Next, we examine changes in MSA mean price, median price, and listing volume, as the number of new positive COVID-19 cases increased. When we estimate the effect of new cases in our two-way fixed effects model (see Table 1), we find a significant, negative relationship with median and mean price.^8^ Nevertheless, the results suggest that on average, rising positive cases are associated with a measurable decline in median posted rental price. More specifically, a 100% increase in positive tests is tied to a $6.52 decline in median price and $5.39 decline in mean price. Keep in mind that a 100% increase occurred multiple times throughout most MSAs during this time period (i.e., going from 5 to 10 cases, then 10 to 20 cases, etc.). Given the rapidly rising positive test rates, the price decline is substantial. Interestingly, we do not find a significant relationship between posting volume and COVID-19 cases. Even though the results show an expected drop in listing volume (about 77 listings) when there is a 100% increase in new cases, we speculate that the wider distribution of listing volumes (standard deviation = 2134.1) is likely to contribute to the lack of statistical significance.

**Table 1.** Change in New Cases and Rental-Related Outcomes (MSA).

	Dependent variable		
	Mean price	Median price	Number of listings
	(1)	(2)	(3)
New cases (logged)	−7.781* (3.836)	−9.411* (3.828)	−77.834 (64.741)
Observations	588	588	588
*R* ^2^	.996	.995	.958
Adjusted *R*^2^	.996	.995	.954
Residual standard error	31.328	31.292	459.060

*Note*. The unit of analysis of these models is week-MSA. The time frame of these models begins on March 15, 2020, and ends on June 1, 2020. The regression models include MSA and week fixed effects. Standard errors are clustered at the MSA level. MSA = metropolitan statistical area.

† *p* < .1. *p < .05. **p < .01.

**Figure 4.**
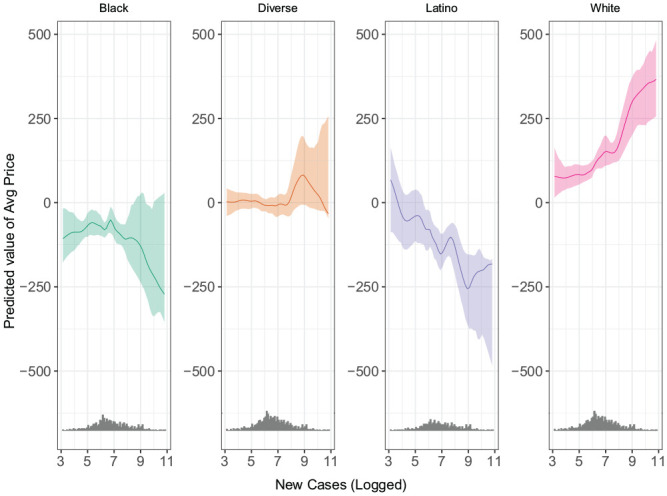
Predicted value of average price by neighborhood type. *Note*. The figure shows the predicted average price by neighborhood type from the two-way fixed models. The predicted average price was derived using Hainmueller et al.’s (2019) method. The predicted values do not show a linear pattern because Hainmueller et al. (2019) does not impose linearity in the interaction models.

We find substantial heterogeneity when we disaggregate the effect of rising cases in a MSA across listings by racial/ethnic makeup. Recall that in these models, we compare weekly average rental prices among listings located in four types of neighborhoods (majority White, Black, Latino, and diverse, which includes all tracts without a clear majority group as well as the only two majority Asian tracts in our data) in each MSA.^9^ We interact our indicators of listing neighborhood type with the number of new cases (logged), treating listings in majority-White neighborhoods as the reference category.^10^ Because our key measure of new cases is logged, directly interpreting the interaction coefficients is difficult. Instead, in Figure 4, we plot the predicted change in price for listings from each type of neighborhood as the number of new cases (logged) increases. Figure 4 makes it clear that as cases rise, prices in Black, Latino, and diverse neighborhoods tend to drop. In majority White neighborhoods (the reference category), prices increase as the case rate rises.

These patterns suggest that the overall negative effect of Covid-19 cases on price we identified in our previous analysis is being driven by listings in Black, Latino, and diverse neighborhoods. The effect appears to be steepest in Latino neighborhoods. Given the stark differences depicted here, it is imperative for future work to identify the mechanisms and underlying structural causes of these disparities.

We do not find evidence of an interaction effect for listing volume. While listing volumes are lower in non-White neighborhoods (see Boeing, 2020), we find no significant changes over time or with the positive case rate. Combined with our aggregate results, this suggests that in general, landlords and property managers did not hold vacancies off the market during this time period. However, the results for mean and median rental price underscore the disproportionate impact of COVID-19. Our findings suggest that the aggregate drop in rental prices across the 49 largest MSAs is being driven by neighborhoods that are not majority White (Table 2).

**Table 2.** Neighborhood Types and Rental-Related Outcomes (MSA).

	Dependent variable					
	Mean price		Median price		Number of listings	
	(1)	(2)	(3)	(4)	(5)	(6)
New cases (logged)	−11.244* (4.578)	27.389** (9.317)	−11.699* (4.655)	19.234* (8.533)	−24.777 (20.590)	−64.171 (62.873)
Black	−229.278** (28.409)	180.957 (127.806)	−193.148** (26.669)	63.401 (134.743)	−1357.052** (140.937)	−1369.239* (514.714)
Latino	−274.906** (36.188)	292.646* (116.013)	−231.898** (34.618)	244.396^†^ (125.000)	−1340.164** (187.874)	−2035.697* (791.367)
Diverse	−136.196** (17.260)	88.669 (72.290)	−111.052** (13.927)	109.617* (41.544)	−958.810** (156.307)	−1421.300* (637.119)
New cases (logged) × Black		−61.320** (21.189)		−38.552^†^ (22.401)		2.966 (72.612)
New cases (logged) × Latino		−83.387** (16.952)		−69.847** (18.049)		101.464 (106.717)
New cases (logged) × Diverse		−33.715** (11.942)		−33.086** (6.622)		69.344 (85.664)
Observations	2,012	2,012	2,012	2,012	2,012	2,012
*R* ^2^	.945	.951	.936	.940	.648	.651
Adjusted *R*^2^	.943	.950	.934	.938	.637	.639
Residual standard error	121.356	114.434	120.583	116.367	612.468	610.454

*Note*. The time frame of these models begins on March 15, 2020, and ends on June 1, 2020. The regression models include MSA and week fixed effects. The baseline for the neighborhood type variable is White neighborhoods. Standard errors are clustered at the MSA level. MSA = metropolitan statistical area.

† *p* < .1. *p < .05. **p < .01.

## Discussion

Segregation exacerbates inequalities as well as the effects of economic and environmental disasters. Indeed, racial residential segregation increased the racially disparate impacts of the foreclosure crisis during the Great Recession (Dwyer & Lassus, 2015; Rugh & Massey, 2010) and multiple studies have documented how segregated neighborhoods are more vulnerable to floods and hurricanes and recover more slowly in their aftermath (Faber, 2015; Fothergill & Peek, 2004; Freudenberg et al., 2009; Fussell, 2015; Pais & Elliott, 2008). The COVID-19 pandemic is likely no different: the economic impacts—which will undoubtedly be long-lasting—have fallen disproportionately on Black and Latino households (Chetty et al., 2020). Here, we have examined the effects of the pandemic on the rental market in the 49 largest U.S. MSAs and found that, while asking rents are down overall, this trend is driven by drops in Black and Latino neighborhoods. In contrast, White neighborhoods experienced price increases in the first few months of the pandemic. There is also a great deal of variation across MSAs, likely caused by distinct mobility patterns, the extent of racial segregation, local COVID-19 related ordinances, and other factors. Future research should certainly explore the why the rental market in some metros has been more or less affected by COVID-19 infection rates.

In these early analyses, we cannot specify the mechanisms by which Black and Latino neighborhoods are more affected. However, recently reported mobility patterns illustrate that the economic impacts of the pandemic are not evenly felt across places. First, mail forwarding requests and aggregated smartphone mobility data indicate residents of central city neighborhoods with higher shares of White residents moved as infection rates increased compared with residents in less White neighborhoods (Paybarah et al., 2020). Those who stayed within their MSA or moved to other MSAs likely moved to neighborhoods with similar demographics to the neighborhoods they left, which could help explain sustained demand in White neighborhoods overall. Second, higher shares of poor and non-White households have experienced job loss and reduced income due to COVID-19 compared to White households (Parker et al., 2020), likely meaning reduced demand and general economic activity in poorer and Black and Latino neighborhoods. Such a reduction in demand could potentially explain some of the reductions in price in non-White neighborhoods. Third, neighborhoods with fewer White residents, as well as poorer neighborhoods, have higher infection rates overall (Nichols et al., 2020; The Stoop, 2020). Future research could examine whether these factors sustained demand for housing in whiter neighborhoods and reduced it in Black, Latino, and diverse neighborhoods.

Our evidence of potentially declining rental market activity in Black and Latino neighborhoods suggests the economic fallout will be harder in these places. While price decreases may be, in some ways, economically beneficial for rental housing market consumers in the short-term, past crises in the housing market have broadly led to more consolidation of rental market housing stock and adverse outcomes for residents of already disadvantaged neighborhoods. It will be critical for future scholarship to examine the medium- and long-term consequences of the short-term market changes we have identified here. Such research will only be possible if we collect the necessary data now. In particular, our analysis highlights the limitations of collecting listing data only twice weekly, as we do not have enough new listings each week to support a tract-level analysis. We hope this article spurs researchers to begin and further strengthen data collection efforts so that such analyses will be possible in the future.

In addition, a looming eviction crisis could further exacerbate the ethnoracial inequalities we identify, as could subsequent waves of rising Covid-19 cases. Eviction moratoria and rental assistance programs are more than warranted and our research also reveals that measures to prevent foreclosure for small-scale property owners are also necessary. Preventing corporate consolidation of housing stock in poor and non-White neighborhoods should be a policy priority. What is clear is that far more research is needed to understand the consequences of the ongoing pandemic. While our preliminary analysis offers more questions than answers, by highlighting the importance of studying the rental market broadly and testing for racial disparities within the rental market in particular, we hope to set the stage for future work.

## Appendix A

### Using Tract-Week as the Unit of Analysis

We do not use Census tract-week as the unit of analysis in our study because many Census tracts do not have listings for every week. If we use tract-week as the unit of analysis, we are going to have a large amount of missing observations. Figure A1 displays the prevalence of missingness in Craigslist data. We randomly selected 100 census tracts and visualize which tract has observation or not. The visualization was done by using Liu and Xu’s panel view. Only a few census tracts in the figure have complete information. Most Census tract have less than half of the observations. Running two-way fixed models with an unbalanced panel like this will likely bias any results.

Even though two-way fixed models on an unbalanced panel are likely to bias the results, we conduct the same two-way fixed models as the main text to show the results are similar regardless of the unit of analysis.

The results in Table A1 are very similar to the results in the main text (Table 2). Listings in White neighborhoods have positive and statistically significant relationship with the logged new COVID-19 cases. However, Black, Latino, and diverse neighborhoods have negative and statistically significant relationship with logged new cases.

**Table A1.** New COVID-19 Cases and Rental-Related Outcomes Interacted With Neighborhood Types: Tract-Week as the Unit of Analysis.

	Dependent variable					
	Mean price		Median price		Number of listings	
	(1)	(2)	(3)	(4)	(5)	(6)
New cases (logged)	2.37 (3.10)	19.03** (5.62)	2.96 (3.07)	18.66** (6.17)	−0.20 (0.20)	0.12 (0.23)
Black	−270.75** (29.59)	134.10^†^ (74.92)	−257.72** (27.75)	118.07 (73.19)	−1.26 (0.77)	0.09 (3.82)
Latino	−344.69** (62.71)	2.19 (134.95)	−330.58** (59.23)	−4.98 (132.04)	0.11 (0.93)	2.68 (3.02)
Diverse	−198.69** (29.72)	27.80 (90.49)	−191.82** (29.09)	25.14 (89.67)	3.27** (0.78)	12.69** (3.19)
New cases (logged) × Black		−56.18** (11.61)		−52.16** (11.24)		−0.20 (0.47)
New cases (logged) × Latino		−47.53* (22.82)		−44.62* (22.23)		−0.37 (0.39)
New cases (logged) × Diverse		−31.93* (13.60)		−30.57* (13.62)		−1.32** (0.38)
Observations	160,115	160,115	160,115	160,115	160,115	160,115
*R* ^2^	.46	.46	.44	.45	.05	.05
Adjusted *R*^2^	.46	.46	.44	.44	.05	.05
Residual standard error	599.12	598.42	612.73	612.13	27.79	27.78

† *p* < .1. **p* < .05. ***p* < .01.

## Appendix B

### Weighting Models Using the Number of Tracts for Each Neighborhood Type

For our models testing for heterogeneity in the association between new COVID-19 cases and rental outcomes, we use four neighborhood types (classified by majority ethnoracial group) in each MSA as the geographical unit of analysis. The shortcoming of choosing this unit of analysis is that it treats each neighborhood type as equal units even though these units are not equal. For example, there are more Census tracts in Atlanta that are White and Black majority than Latino majority. However, our main models treat them as equal units. To make sure this analytical choice does not bias our results, we include the number of tracts as weights in the two-way fixed effect models to give higher weights to neighborhood types that have more Census tracts and population. To explain this weighting scheme using the Atlanta example, White (131 tracts) and Black (96 tracts) neighborhoods will get higher weights than Latino neighborhoods (11 tracts).

The results presented in Table A2 shows similar result to Table 2. In fact, the differences across neighborhood types are more pronounced than Table 2.

**Table A2.** New COVID-19 Cases and Rental-Related Outcomes Interacted With Neighborhood Types: Weighting by the Number of Census Tracts in Each Neighborhood Type-Week.

	Dependent variable					
	Mean price		Median price		Number of listings	
	(1)	(2)	(3)	(4)	(5)	(6)
New cases (logged)	−5.82 (4.18)	16.02** (5.69)	−6.85* (3.29)	9.79* (4.84)	−25.11 (47.37)	−33.25 (65.01)
Black	−290.33** (42.00)	259.54* (100.68)	−243.45** (34.84)	114.88 (97.17)	−1287.48** (169.82)	−982.26^†^ (563.86)
Latino	−347.49** (67.74)	173.99 (116.62)	−287.63** (55.78)	100.53 (116.55)	−1049.72** (281.84)	−1408.80* (663.94)
Diverse	−180.32** (25.14)	74.74 (81.15)	−138.84** (17.48)	89.57 (55.55)	−825.48** (196.90)	−1087.39^†^ (594.20)
New cases (logged) × Black		−76.25** (16.85)		−49.85** (16.25)		−41.00 (81.95)
New cases (logged) × Latino		−71.21** (20.33)		−53.02** (18.97)		48.32 (88.48)
New cases (logged) × Diverse		−36.02** (12.34)		−32.16** (7.86)		36.33 (73.78)
Observations	2,012	2,012	2,012	2,012	2,012	2,012
*R* ^2^	.97	.98	.97	.98	.77	.77
Adjusted *R*^2^	.97	.98	.97	.98	.77	.77
Residual standard error	904.00	829.49	787.49	743.45	5248.85	5244.27

† *p* < .1. **p* < .05. ***p* < .01.

## Appendix C

### Measuring the Number of COVID-19 Cases on a Per Capita Basis

The absolute number of COVID-19 cases is likely to differ by the population size of each MSA. More populous MSAs are likely to have higher case counts than less populated ones. In this section, we reestimate our models in Tables 1 and 2 with logged COVID-19 cases per capita to show that our results are robust to population size.

**Table A3.** Table 1 Models Using Per Capita Measures.

	Dependent variable		
	Mean price	Median price	Number of listings
	(1)	(2)	(3)
New cases per capita (logged)	−7.781* (3.836)	−9.411* (3.828)	−77.834 (64.741)
Observations	588	588	588
*R* ^2^	.996	.995	.958
Adjusted *R*^2^	.996	.995	.954
Residual standard error	31.328	31.292	459.060

† *p* < 0.1. **p* < .05. ***p* < .01.

**Table A4.** Table 2 Models Using Per Capita Measures.

	Dependent variable					
	Mean price		Median price		Number of listings	
	(1)	(2)	(3)	(4)	(5)	(6)
New cases per capita (log)	−11.244* (4.578)	8.954 (8.670)	−11.699* (4.655)	10.273 (8.198)	−24.777 (20.590)	−62.820 (61.131)
Black	−229.278** (28.409)	−405.636** (119.964)	−193.148** (26.669)	−361.395** (110.305)	−1357.052** (140.937)	−243.840 (597.280)
Latino	−274.906** (36.188)	−788.859** (157.413)	−231.898** (34.618)	−722.165** (178.226)	−1340.164** (187.874)	−1162.109 (781.183)
Diverse	−136.196** (17.260)	−222.696* (101.451)	−111.052** (13.927)	−263.613** (72.602)	−958.810** (156.307)	−938.223^†^ (532.634)
New cases per capita (log) × Black		−22.935 (14.346)		−21.794 (13.032)		147.743^†^ (78.927)
New cases per capita (log) × Latino		−65.527** (19.907)		−62.578** (22.397)		23.182 (106.354)
New cases per capita (log) × Diverse		−11.214 (12.204)		−19.779* (8.703)		2.669 (78.351)
Observations	2,012	2,012	2,012	2,012	2,012	2,012
*R* ^2^	.945	.949	.936	.940	.648	.655
Adjusted *R*^2^	.943	0.947	.934	.938	.637	.644
Residual standard error	121.356	117.430	120.583	117.205	612.468	606.738

† *p* < .1. **p* < .05. ***p* < .01.

## Appendix D

### Table 2 With Additional Covariates

In this section, we present models that include covariates that might potentially confound the association between ethnoracial composition and rental prices. In the models presented in Table A5, we include housing density, occupational composition, and tenant mobility. These measures are obtained from 2018 ACS 5-year pooled data.

**Table A5.** Table 2 Models Including Additional Covariates.

	Dependent variable					
	Mean price		Median price		Number of listings	
	(1)	(2)	(3)	(4)	(5)	(6)
New cases (logged)	−9.002^†^ (4.898)	21.418* (8.511)	−10.284* (4.905)	15.853^†^ (8.863)	−12.549 (21.867)	−117.127^†^ (65.467)
Black	−215.374** (37.838)	67.883 (118.539)	−184.963** (37.322)	−8.321 (128.343)	−1219.583** (179.224)	−2044.955** (630.964)
Latino	−174.735** (51.987)	352.065* (135.183)	−166.454** (50.309)	289.830* (138.959)	−934.565** (302.011)	−2115.866** (712.797)
Diverse	−125.946** (28.829)	33.341 (68.581)	−105.040** (26.960)	76.815 (47.059)	−849.953** (223.744)	−1875.836** (698.914)
Pct manufacturing	−9.494** (2.983)	−9.393** (2.625)	−6.268** (2.323)	−6.349** (2.059)	−31.852* (15.101)	−32.375* (14.697)
Pct no move	1.054 (1.329)	1.317 (1.239)	0.731 (1.185)	1.038 (1.141)	0.042 (5.301)	−0.600 (5.148)
Housing density (logged)	0.010 (0.007)	0.004 (0.007)	0.006 (0.006)	0.001 (0.006)	0.075 (0.050)	0.094* (0.046)
New cases (logged) × Black		−43.799* (19.612)		−27.980 (22.086)		127.424 (84.909)
New cases (logged) × Latino		−78.088** (19.596)		−67.483** (20.618)		177.214^†^ (98.467)
New cases (logged) × Diverse		−24.808* (9.990)		−28.226** (7.325)		156.265 (95.776)
Observations	2,012	2,012	2,012	2,012	2,012	2,012
*R* ^2^	.952	.956	.939	.943	.677	.684
Adjusted *R*^2^	.950	.955	.937	.941	.666	.673
Residual standard error	114.098	108.560	117.602	113.909	587.658	581.271

† *p* < .1. **p* < .05. ***p* < .01.

## Notes

1. In most cases, Craigslist sites for MSAs closely match the census definition. However, some are distinct. Craigslist, for example, puts both the San Francisco-Oakland-Hayward, CA MSA and the San Jose-Sunnyvale-Santa Clara, CA MSA in one site titled SFBay. We refer to this site as the San Francisco Bay Area. Similarly, while the Census treats Miami, Fort Lauderdale, and West Palm Beach, FL as one MSA, in May of 2017 when we began data collection, each of these areas had its own Craigslist site. We combine all unique listings from each site and refer to them as Miami. Lastly, the Craigslist site for Los Angeles covers Los Angeles County rather than the Los Angeles-Long Beach-Anaheim MSA, which includes Orange County. Orange county is not included in our analyses.

2. Including duplicates increases our statistical power but does not substantively change any of our findings. We use the reduced, nonduplicate data set to produce more conservative estimates.

3. Social explorer retrieves county data from the *New York Times* COVID-19 database and aggregates them up to the MSA-level.

4. The absolute number of COVID-19 cases vary depending on the population size of each metro. We reestimate our models with logged COVID-19 cases per capita to demonstrate that our results are robust to population size. The results are reported in Tables A3 and A4 (Appendix C).

5. Our sample for the two-way fixed effects models starts on March 15, the third week of March and right after national emergency was declared. Most MSAs did not have any confirmed cases before March 15.

6. Asian majority neighborhoods are classified as diverse neighborhoods because there are only two Asian majority neighborhoods across the MSAs in our sample and because of the high margins of error on tract-level ACS data for ethnic groups with low representation.

7. Even though we recognize using tract-week as the unit of analysis would likely to bias our results, reestimate our models examining within-MSA heterogeneity using tract-weeks and find substantively similar results. The results are presented in Table A1 (Appendix A).

8. The results for mean price are not robust to different degree-of-freedom adjustment methods. The clustered standard errors vary from 3.8179 to 3.8724. The lowest *p* value is .042 that is derived from block bootstrapped standard errors calculated by Stata’s *reghdfe*, We obtain the highest *p* value (.0501) from standard errors calculated by Stata’s *reghdfe*.

9. In the appendices, we reestimate these models using (1) tracts (see Table A1, Appendix A) and (2) weighted models that use the number of tracts for each neighborhood type (see Table A2, Appendix B). In both cases results are substantively similar.

10. There are covariates that might potentially confound the association between ethnoracial composition and rental prices such as housing density, occupational composition, and tenant mobility. In Table A5 (Appendix D), we present regression results that include these covariates. Our results are robust to the inclusion of these control measures.
